# Fiber-Shaped Triboiontronic Electrochemical Transistor

**DOI:** 10.34133/2021/9840918

**Published:** 2021-04-26

**Authors:** Jinran Yu, Shanshan Qin, Huai Zhang, Yichen Wei, Xiaoxiao Zhu, Ya Yang, Qijun Sun

**Affiliations:** ^1^Beijing Institute of Nanoenergy and Nanosystems, Chinese Academy of Sciences, Beijing 101400, China; ^2^School of Nanoscience and Technology, University of Chinese Academy of Sciences, Beijing 100049, China; ^3^Department of Materials Science WW-4, LKO, University of Erlangen-Nuremberg, Martensstrasse 7, 91058 Erlangen, Germany; ^4^Center on Nanoenergy Research, School of Physical Science and Technology, Guangxi University, Nanning 530004, China; ^5^Beijing Institute of Fashion Technology, Beijing 100029, China

## Abstract

Contact electrification-activated triboelectric potential offers an efficient route to tuning the transport properties in semiconductor devices through electrolyte dielectrics, i.e., triboiontronics. Organic electrochemical transistors (OECTs) make more effective use of ion injection in the electrolyte dielectrics by changing the doping state of the semiconductor channel. However, the mainstream flexible/wearable electronics and OECT-based devices are usually modulated by electrical signals and constructed in conventional geometry, which lack direct and efficient interaction between the external environment and functional electronic devices. Here, we demonstrate a fiber-shaped triboiontronic electrochemical transistor with good electrical performances, including a current on/off ratio as high as ≈1286 with off-current at ~nA level, the average threshold displacements (*D*_th_) of 0.3 mm, the subthreshold swing corresponding to displacement (SS_D_) at 1.6 mm/dec, and excellent flexibility and durability. The proposed triboiontronic electrochemical transistor has great potential to be used in flexible, functional, and smart self-powered electronic textile.

## 1. Introduction

Contact electrification-activated triboelectric potential, which can be readily generated from a triboelectric nanogenerator (TENG), offers an efficient way to tuning the transport properties in semiconductor devices and artificial afferents [1]. It has also been developed toward versatile applications derived by mechanical behavior [2], such as logic devices [3, 4], multifunctional sensory devices [5], tribotronic memories [6], intelligent flexible/wearable sensors [7–11], and mechanoplastic neuromorphic devices [12–14]. To explore more extensive research and development, further improvements on the gating efficiency of triboelectric potential by effectively coupling TENG and transistor device have become urgent so as to enhance the electrical properties of tribotronic transistors. Generally, the dielectrics in the transistor act as the critical capacitive layer and provide a valid approach on the modulation of gating properties. Polymer electrolytes, e.g., ion gels, are preferred to be used as gate insulator materials in field effect transistors. The electric double layer (EDL) formed at the electrolyte/semiconductor interface provides a very high electric field coupled to the semiconductor channel. It can also suppress the short-channel effect at a lower applied gate voltage and efficiently reduce the device power consumption [15–17]. The ion gel has been successfully verified in a triboiontronic transistor of MoS_2_ [18], which has shown excellent electrical performances and figure of merits, including the current on/off ratio at seven orders of magnitude, proper tribotronic threshold value at 75 *μ*m, and good subthreshold swing of 20 *μ*m/dec.

Different from EDL transistors, organic electrochemical transistors (OECTs) make more effective use of ion injection because the ions can penetrate the semiconductor layer and lead to the change of the doping state in the whole channel [19]. One of the most promising materials so far for OECTs is the low-bandgap conducting polymer poly(3,4-ethylenedioxythiophene) (PEDOT), owing to its high conductivity, environmental stability, and water dispersibility in the form of poly(3,4-ethylenedioxythiophene)/poly (styrene sulfonic acid) (PEDOT:PSS) [20–22]. Compared with the traditional electrochemical transistors, fiber-based OECTs have attracted great attentions due to the advantages of adjustable fabrication, good air stability, low driving voltages, compatibility with flexible substrate, and low cost over large areas [23–28]. By embedding the electronic functional components into fabrics, fiber-based OECTs facilitate the rapid development of functional circuits on textile materials and provide various compelling applications including dynamic health monitoring, wearable smart computers, and light-emitting diodes (LEDs) [29]. However, the mainstream flexible/wearable fibrous electronics and OECT-based devices are usually modulated by electrical signals, which lack direct interaction between the external environment and electronic devices. Besides, a key step to implementing intelligent e-textiles is to pair the fabrication process of both functional components and energy devices with compatible textile production. The fibrous OECTs combine functional fibers with logic transistor devices to implement the reversible process of doping/dedoping of conducting polymers in fibrous structure, providing an easier route for electronic devices to be directly woven into fabrics and facilitate their integration with TENG cloths.

Here, we propose a fiber-shaped triboiontronic electrochemical transistor gated by a woven-structured TENG cloth. Triboelectric potential can be coupled to the PEDOT:PSS channel of the fiber-shaped OECT to induce a reversible doping/dedoping process and modulate the output current via hybrid ion gel dielectrics (70 wt% and 90 wt%). The fiber-shaped OECT working in a depletion mode shows excellent electrical performances, including a high current on/off ratio of 4000, low gate leakage current of less than 4.2 nA, and good bending and cycle stability. Thanks to the high gating efficacy by triboelectric potential through EDLs with ultrahigh capacitance, the fiber-shaped triboiontronic electrochemical transistor also shows comparable electrical properties with a high current on/off ratio (≈1286), low off-state current, proper threshold displacement (*D*_th_, ~0.3 mm), and low tribotronic subthreshold swing (SS_D_, ~1.6 mm/dec). It can also be readily integrated into a 3 × 3 device array and driven by the woven-structured TENG globe gate, working as a flexible, functional, and intelligent self-powered electronic textile. Furthermore, a woven logic inverter based on the proposed fiber-shaped triboiontronic electrochemical transistor has been achieved. The structure of this device can be used to control the on/off-state of the LED. The self-powered electronic textile based on fiber-shaped triboiontronic OECT can directly utilize the triboelectric potential of the woven-structured TENG to tune the doping/dedoping in the conducting polymer, which realizes the effective combination of mechanical actions and electrochemical reactions in OECT for the first time. It has extensive prospect in the field of self-powered devices and wearable human-machine interface.

## 2. Results

**Figure 1:**
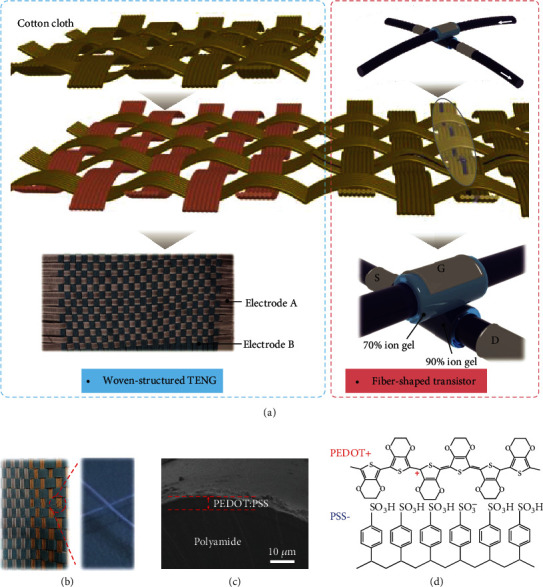
Schematic illustration of the fibrous triboiontronic electrochemical transistor. (a) The schematic diagram of fiber-shaped triboiontronic OECT, which consists of a woven-structured TENG and a fiber-shaped OECT. (b) The optical image of the triboiontronic electrochemical transistor. (c) The SEM image of the PEDOT:PSS film. The thickness of the PEDOT:PSS film is evaluated to be 6 *μ*m. (d) The chemical structure of PEDOT^+^ and PSS^−^.

Schematic illustration of the self-powered electronic cloth based on a fiber-shaped triboiontronic electrochemical transistor is depicted in Figure 1(a), which contains two primary components: a fiber-shaped OECT based on PEDOT:PSS and a freestanding woven-structured TENG cloth in a contact separation mode to scavenge electric energy from human motions (optical image in Figure 1(b)). The fabrication process of the fiber-shaped OECT is illustrated in Figure S1. Firstly, a thin PEDOT:PSS layer is coated on the surface of the whole nylon fiber through the dip drop process. The thickness of the PEDOT:PSS film is evaluated to be 6 *μ*m as shown in SEM image of Figure 1(c). Then, 70 wt% ion gel is cast around the fiber to define the channel length, which is controlled to be 1 mm. And the channel width is equal to *π* · *D*, where *D* is the diameter of the fiber. The 90 wt% ion gel is further prepared to cover on the PEDOT:PSS channel to improve the gating efficiency. The electrical properties of the OECTs based on 70 wt% and 90 wt% ion gels are compared in Figure S2. The OECT with 70 wt% ion gel shows low gate leakage current but weak electric field control performance, while the OECT with 90 wt% ion gel has a larger switching ratio but higher gate current. Silver pastes are patterned on the 70 wt% ion gel and both ends of the PEDOT:PSS channel, working as the gate and source drain electrodes, respectively. Then, the prepared two fibers are manually attached at the ion gel anchor position and assembled in a crossbar geometry to achieve the fiber-shaped OECT (detailed procedures in Materials and Methods). Finally, the triboiontronic electrochemical transistor is obtained by the combination of the woven-structured TENG cloth and the fibrous transistor. Notably, the hybrid ion gel components including the attached 70 wt% and 90 wt% ion gel layers are elaborately prepared as the dielectrics for the fibrous triboiontronic transistor. The 70 wt% ion gel mainly functionalizes a blocking dielectric layer to decrease the gate leakage current, while the 90 wt% ion gel is capacitively coupled and aimed at maintaining the dielectric capacitance at a comparable high value with the pure 90 wt% ion gel to ensure the high gating efficiency in the electrochemical devices [5]. For the transistor channel, the semiconductor PEDOT^+^ is *p*-type doped that the positive polaron on the PEDOT chain (standing for a mobile hole) can jump from one chain to another. PEDOT:PSS has hole conduction due to charge compensation by the immobile sulfonate anions on the PSS chain. Chemical structures of PEDOT and PSS are shown in Figure 1(d) [19]. The relationship between the resistance of the PEDOT:PSS-coated fibers and their specific length is depicted in Figure S3, representing that a uniform thin film of PEDOT:PSS is prepared by the dip drop process.

**Figure 2:**
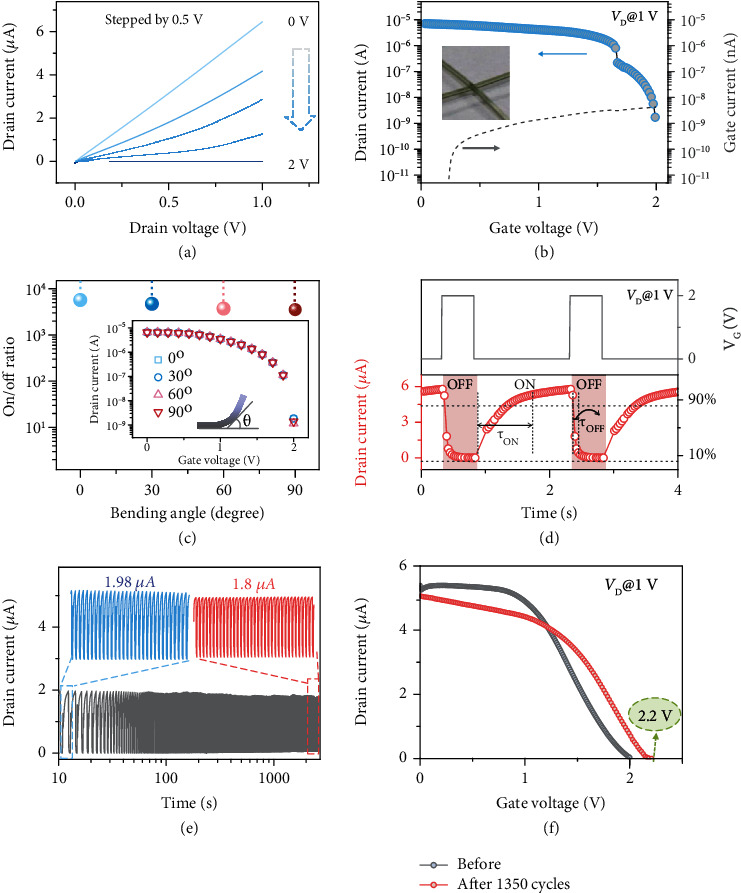
Electrical characterization of the fiber-shaped OECT. (a) Output curves of the fiber-shaped organic electrochemical transistor. The typical output curves (*I*_D_-*V*_D_) of the fiber-shaped OECT under *V*_D_ sweeping (0~1 V) at different *V*_G_ values (0~2 V) with a step of 0.5 V. The output curve shows a decline in channel conductance with increasing *V*_G_. (b) The drain current (*I*_D_) and gate leakage current (*I*_G_) in the logarithmic scale vs. gate voltage (*V*_G_) of the fiber-shaped OECT. Here, the *V*_G_ sweeps from 0 to 1 V when *V*_D_ = 1 V. The current on/off ratio reaches 4000. The gate leakage current (*I*_G_) is reduced to several nA. (c) The on/off ratios and transfer curves of the fiber-shaped OECT under different bending angles: 0°, 30°, 60°, and 90°. (d) The real-time drain current and (e) stability test of the device triggered by 2 V square wave voltage pulse. (f) Transfer curves of the fiber-shaped OECT before and after 1350 cycles.

Excellent transistor performances with respect to drain current (*I*_D_) *vs.* gate voltage (*V*_G_) and drain voltage (*V*_D_) modulation in the fiber-shaped OECT are the prerequisite for the operation of the triboiontronic electrochemical transistor. As shown in Figure 2(a), it shows typical output behaviors of a PEDOT:PSS-based OECT working in a depletion mode under different *V*_G_ values (0~2 V) with a step of 0.5 V. Output curves represent a decrement in channel conductance with increased *V*_G_. When the gate voltage is zero, the conducting polymer is dominated by holes and contributes to a high drain current, and the transistor is working in the ON state. When a positive gate voltage is applied, the holes are compensated by cations (generated from ion gel), and the transistor reaches the off state [30]. The current level in the on state (*V*_G_ = 0 V) is ~6.5 *μ*A, while the current level in the reduced off state is ~5 nA (*V*_G_ = 2 V), which results in an on/off ratio of 1300. The corresponding transfer curve of the fabricated device (*V*_D_ = 1 V) is shown in Figure 2(b), also exhibiting typical depletion characteristics with a current on/off ratio of 4000. The gray dotted line in Figure 2(b) is the gate leakage current, which is less than 4.2 nA. Figure S4 gives the real-time (*I*-*t*) test of the fiber-shaped OECT under different gate voltages (0~2 V). The drain current decreases as the gate voltage increases from 0 to 2 V, which is consistent with the transfer curve. For wearable self-powered e-textiles, the capability to withstand deformation or harsh bending status is an important requirement. The flexibility test is conducted as shown in Figure 2(c). The on/off ratio shows a slight decrement, but no obvious change in corresponding transfer curves (inset of Figure 2(c)) can be found when the fiber-shaped OECT is bent at different angles (*θ* = 0° ~ 90°). These results ensure the proper operation state of the electrochemical transistor under bending states.

The above excellent electrical characteristics of the fiber-shaped OECTs motivate us to further pursue the dynamic electrical properties of these devices. The electrochemical response time determined by the dedoping/doping process is related to the volume of channel material and its contact area with the ion gel. It is recorded by evolution of the *I*_D_*vs.* time after applying a square wave voltage pulse to the gate electrode. Obviously, the switching speed of our fiber-shaped OECT is relatively high as evidenced by the drain current transient response in Figure 2(d), which indicates that a complete current on/off period takes about 1.8 s. The on/off response time of the fiber-shaped OECT, defined as the time interval associated with the current variation from 10% to 90% of the maximum drain current, is calculated to be 820 ms (*τ*_ON_) and 135 ms (*τ*_OFF_), respectively. This may be attributed to the differences of the ion mobility in ion gel dielectrics during the turning-on and turning-off process in the OECT with same channel dimension. Increasing the effective contact area between PEDOT:PSS and ion gel can permit considerably faster dedoping of the transistor channel and corresponding response time. Notably, as PEDOT:PSS is susceptible to moisture, relevant humidity influence on the fiber-shaped devices is difficult to be investigated. By introducing a passivation layer or hydrophobic coating materials, the influence of humidity on PEDOT:PSS can be reduced, and the humidity stability of the device may be greatly improved (Supplementary Materials).

The effect of periodic input voltage pulses on device stability has also been considered. The fiber-shaped OECT can be operated for over 1350 cycles without showing any sign of serious degradation (Figure 2(e)). The device shows excellent stability and retains 90.8% of the original *I*_D_ value after 1350 cycles, which can satisfy the specific requirements for durability in practical applications. Transfer characteristics after 1350 cycles in Figure 2(f) show an increase in the off-state current, indicating a decrease in modulation ability of the devices upon cycling. This is probably due to the trapping of bulky EMIM^+^ ions during the turning-off process. It is worth noting that the current on/off ratio can reach the initial value of 1187 with *V*_G_ increased to 2.2 V. Therefore, the demonstrated short switching time and maintained high on/off ratio during flexibility and cycling tests qualify that the fiber-shaped OECTs are suitable to be integrated in soft woven-structured electronic systems and accomplish corresponding applications.

**Figure 3:**
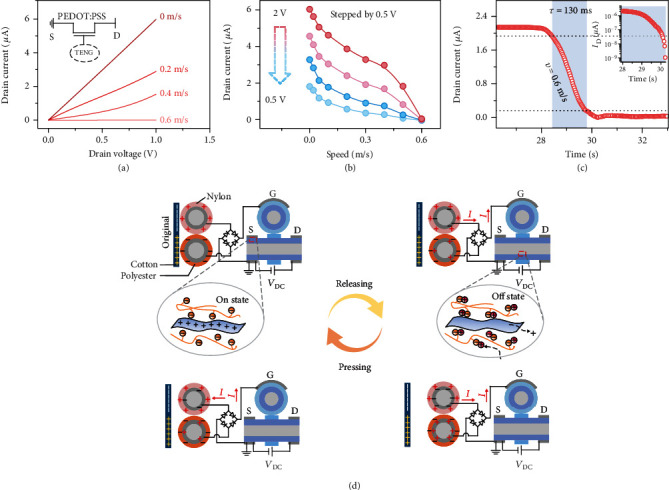
Characteristics of the fibrous triboiontronic electrochemical transistor. (a) Output curves (*I*_D_-*V*_D_) of the tribotronic fiber-shaped OECT under different contact speeds of TENG. The output curve shows a decline in channel conductance with increased contact speeds. (b) Transfer curves (*I*_D_-*v*) of the triboiontronic fiber-shaped OECT under different *V*_D_ values (2~0.5 V). (c) Switching characteristic of the triboiontronic fiber-shaped OECT. The device transformed from an on state to an off state with the contact speed of 0.6 m/s and *τ*_OFF_ = 130 ms. (d) Working mechanism of the triboiontronic fiber-shaped OECT.

For the fiber-shaped triboiontronic electrochemical transistor, the relationship between *I*_D_ and contact separation frequency of the fabric TENG is systematically studied. The contact frequency can be precisely controlled by a linear motor.  Figure 3(a) shows the output performance of the triboiontronic electrochemical transistor under different contact speeds of the woven-structured TENG without external *V*_G_. *I*_D_ decreases from 5.97 *μ*A to 4.64 nA with the contact speed of the woven-structured TENG varying from 0 to 0.6 m·s^−1^ (*V*_D_ = 1 V). Under the variation range of the contact speed, the current on/off ratio reaches as high as ≈1286 with off-current at ~nA level, which is very important for the application of logic devices. In order to present the triboelectric potential-driven output and transfer characteristics of the PEDOT:PSS-based fiber-shaped triboiontronic electrochemical transistor more precisely, *I*_D_s under more detailed contact separation speed intervals of the fabric TENG are added in Figure S5.  Figure 3(b) shows the corresponding transfer characteristics of the fiber-shaped triboiontronic transistor at a *V*_D_ of 2, 1.5, 1, and 0.5 V, which are extracted from Figure S5. It is observed that the *I*_D_ is decreased with the increased contact speed, which will be discussed later in the working mechanism. Dynamic switching characteristics of the fibrous triboiontronic transistor OECT are also evaluated in Figure 3(c), showing that the device turning from the on state to off state requires 130 ms at a contact separation speed of 0.6 m/s. Based on above results, the fibrous triboiontronic device has the similar output and transfer characteristics with the OECT modulated by the external gate voltage, which demonstrates that the mobile hole transport in the channel can be effectively modulated by the triboelectric potential of the woven-structured TENG instead of the conventional applied gate voltage. Therefore, the triboelectric potential created by TENG can modulate the drain current as an equivalent gate voltage. The transfer curves and switching ratios of the OECT driven by the TENG in different bending states (*θ* = 0° ~ 90°) have also been given, as shown in Figure S7. The on/off ratio is close to 10^3^ and only slightly declines with the increased bending angles. The flexibility of the fiber-shaped triboiontronic OECT further ensures its potential application in wearable electronic fabrics.

The working mechanism of the fibrous triboiontronic electrochemical transistor is schematically illustrated in Figure 3(d). At the initial state, the freestanding layer of a cotton cloth is brought into full contact with the TENG cloth, which stays in the charge-balance state without any voltage outputs. Contact electrification will occur because the electronegativity of the polyester, nylon, and cotton cloth is different from each other. The triboelectric effect will render nylon and polyester surface with positive and negative charges and render the contacted positions of the cotton cloth with nylon and polyester fabric with negative and positive charges, respectively. Corresponding redox states of the PEDOT:PSS channel in the fiber-shaped OECT are also shown in detail. Driven by a vertical force, the freestanding cotton cloth will contact and separate from the TENG periodically and generate positive gate bias applied to the fiber-shaped OECT after rectification through a bridge rectifier. At the initial state, PEDOT:PSS is hole dominated and will show a current flow when the source drain voltage is applied. In the absence of a triboelectric potential, a hole current will flow in the channel (i.e., the on state). When the cotton cloth starts to move apart from the TENG cloth, an electric potential difference is produced, which drives electrons to flow from the polyester electrode to the nylon electrode through an external circuit to balance the generated triboelectric potential. An induced positive triboelectric potential can be applied to the fiber-shaped OECT and drives the ion gel cations (i.e., [EMIM]^+^) to inject into the channel to compensate for the anions (PSS^−^). As a result, the number of holes in the channel decreases and the film is dedoped due to that the holes extracted at the drain are not replenished at the source. This process leads to a drop in the drain current, and the device turns to the off state. Repeated contact separation actions lead to an alternating output, which can be converted into one-direction positive output by the bridge rectifier and effectively coupled to the transistor. Accordingly, the relative displacement, contact separation speed, and acceleration can all decide the output of TENG cloth, which will be readily coupled to the fiber-shaped OECT and influence the doping state of the semiconductor channel.

**Figure 4:**
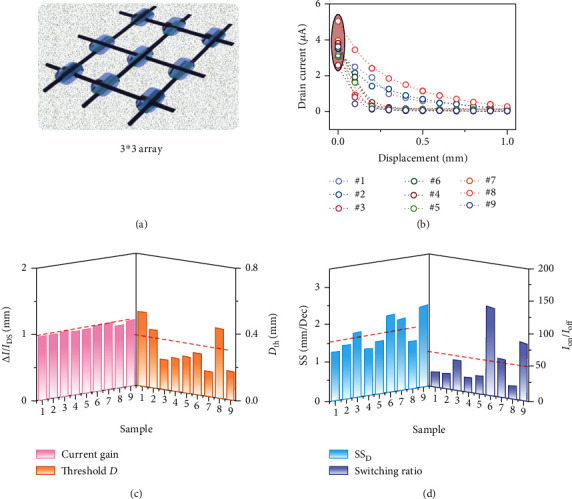
Fibrous triboiontronic device array. (a) Schematic diagram of the 3 × 3 device array based on the triboiontronic fiber-shaped OECT. (b) Transfer curves (*I*_D_-*D*) of nine samples at *V*_D_ = 1 V. (c) Statistics of current gains and threshold displacements (*D*_th_) for nine samples. (d) Statistics of the subthreshold swing for displacement (SS_D_) and switching ratio for nine samples.

A smart self-powered electronic cloth or textile is generally woven or knitted. At each crisscross point, it is possible to integrate one fiber-shaped OECT to construct a transistor array and be actively driven by another TENG cloth.  Figure 4(a) shows the schematic diagram of the 3 × 3 transistor array based on the fibrous electrochemical transistors. By changing the distance between the cotton cloth and TENG cloth, different output curves (*I*_D_-*V*_D_) of the nine transistor devices are obtained, as shown in Figure S6. And the corresponding transfer curves (*I*_D_-*D*, where *D* is the mechanical displacement of the TENG cloth) are extracted in Figure 4(b). The transfer characteristics of the device at each node have shown the same trend and remained at a similar output level. It should be noted that the separation time to reach each distance is guaranteed to be the same, which is consistent with the speed parameters tested above. The displacement *D* is selected as a parameter to more intuitively evaluate the characteristics of the 3 × 3 device array driven by mechanical actions of the TENG cloth. The corresponding figure of merits of the fiber-shaped triboiontronic electrochemical transistor, including current gain, *D*_th_, SS_D_, and switching ratio, has also been elaborately investigated. The current gain levels of nine samples are calculated in the left panel in Figure 4(c), which is close to 100%. The threshold displacements *D*_th_ of nine samples are extracted from transfer curves, as shown in the right panel in Figure 4(c). The average *D*_th_ of the nine transistors at the crisscross nodes is ~0.3 mm. The SS_D_ and switching ratio of each node transistor are shown in Figure 4(d). According to the equation of SS_D_ = *d*_D_/log_10_(*I*_D_), all the extracted SS_D_ of different samples are similar at ~1.6 mm/dec. The achieved switching ratios only have two orders of magnitude (lower than the previous measurement of one single fibrous triboiontronic transistor), which may be due to the potential risk of the leakage current increment after connecting all the devices in the array. The on-state current of all transistors is maintained at a high level of ~3 to 5 *μ*A, which means that the demonstrated triboiontronic OECT-based textiles still have great potential for practical application.

This smart self-powered electronic textile is further exploited to demonstrate the performance of digital or analog electronics. A logic inverter, which accepts a digital Hi or Lo signal and outputs the opposite signal, is the simplest but critical logic gates to construct the complex and advanced digital logic circuits. In the configuration of complementary logic circuits, the inverters consist of an *n*-type and a *p*-type transistor in series to share the common gate, which allows for a low-power logic device with good noise margins andgain. However, it is only achievable when both *n*- and *p*-type transistors have comparable mobilities. As is known, there is a relative dearth of *n*-type OECT materials, and those that are available cannot match the high mobility of *p*-type PEDOT:PSS, which necessitates the use of unipolar logic or analog circuits. In addition to improving the performance and simplifying the design of digital logic, fiber-shaped triboiontronic devices promise additional and good electromechanical performance for analog circuits.

**Figure 5:**
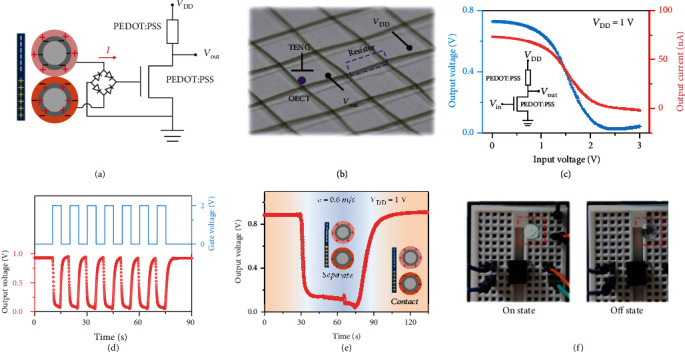
Fiber-shaped triboiontronic logic device based on the electrochemical transistor. (a) Circuit diagram and (b) optical image of the logic inverter based on the triboiontronic fiber-shaped OECT. (c) Typical voltage/current transfer characteristics of the inverter. (d) The real-time output voltage of the inverter triggered by gate voltage (*V*_G_ = 2 V). (e) The real-time output voltage of the inverter triggered by TENG with contact separation speed at 0.6 m/s. (f) The green LED can be switched on/off directly by the TENG.

As a prototypical circuit, a fiber-shaped triboiontronic electrochemical transistor as a common-source amplifier is implemented to construct the logic gate by placing the drain in series with a load resistor (Figure 5(a)). The coated polymer with lower conductivity is used as the load resistor and the cables, providing an easy route to weaving the analog circuit directly into fabrics. The corresponding optical image is photographed in Figure 5(b). The voltage/current transfer characteristics of the analog circuits are depicted in Figure 5(c) at *V*_DD_ = 1 V. The curves in Figure 5(d) show the electrical inputs/outputs of the device, clearly demonstrating high modulation on the switching of the logic device between on and off states (switching to the off state only occurs when *V*_G_ is applied). Similarly, the output voltages of the woven-structured TENG at a contact separation speed of 0.6 m·s^−1^ can be used as input signals of the analog circuit and implement the switch function as shown in Figure 5(e). The proper operation of the triboiontronic logic gate demonstrates that the output voltage created by TENG can also readily modulate the output voltages in analog circuits. A LED is further connected in the proposed analog circuit in series to better demonstrate the application of the smart self-powered e-textile. As shown in Figure 5(f), without mechanical actions on the woven-structured TENG cloth, the LED is turned on at *V*_DD_ = 1 V because the fiber-shaped OECT is in the on state. When the TENG is subject to the contact separation action, the LED is turned off because the fiber-shaped OECT is cut off by the triboelectric potential derived from the mechanical action.

## 3. Conclusion

In conclusion, based on the triboelectric potential modulation on the electrochemical reaction, a fiber-shaped triboiontronic electrochemical transistor has been proposed for the first time. Fibers are now available in textile-compatible form, and their integration with the woven-structured TENG and garments is deserved to be pursued, which can be enabled by this work. Parallel electrical performances include the high current on/off ratio (~1286), low off-current (several nA), proper threshold displacement (~0.3 mm), and steeper tribotronic subthreshold swing (~1.6 mm/dec). Furthermore, the logic inverter based on the proposed fibrous triboiontronic transistor has been realized. The device can control the switch state of LED by mechanical signal. Using simple and elegant solutions, the interwoven smart self-powered electronic textile with OECT on fibers can be achieved for local signal acquisition and early conditioning in e-textiles. This provides a possibility for the revolution of the existing wearable electronic technology.

## 4. Materials and Methods

### 4.1. Fabrication Process of the Woven-Structured TENG

The raw materials for the woven-structured TENG are merchandise polyester fabric (60 *μ*m thick), nylon fabric (40 *μ*m thick), and conductive Ni-coated fabric (125 *μ*m thick). Conductive Ni-coated fabric is cut to straps (3 mm × 12 cm), and the nylon fabric and polyester fabric are also cut into strips (5 mm × 12 cm). Then, one Ni-coated belt is pasted onto the mezzanine of two polyester fabric strips using double-sided adhesive. Here, the width and length of the Ni-coated belts are less than those of polyester fabric so that it can be fully wrapped, avoiding leakage of electricity. The fabrication process of the nylon electrode is similar to that of the polyester electrode. Finally, a 10 cm × 10 cm TENG cloth was woven by using the prepared nylon electrodes and polyester electrodes as longitudes and altitudes, respectively.

### 4.2. Preparation of the Self-Powered e-Textile Employed Fiber-Shaped OECT

We use a high-conductivity formula of PEDOT:PSS containing 90% by weight of PEDOT:PSS aqueous solution (1.4 wt% in water, Adamas) and 10% glycerol. Merchandise polyamide fibers with a diameter of 200 *μ*m are used as the starting substrate for the synthesis of fiber-shaped OECT. The coating is carried out using a vertical flow of the PEDOT:PSS in the form of drops under the influence of gravity down to the exterior of the nylon fibers. The ion gel solution, a mixture of the 1-ethyl-3-methylimidazolium bis(trifluoromethyl sulfonyl) imide ([EMIM][TFSI]) ion liquid, the poly(ethylene glycol) diacrylate (PEGDA) monomer, and the 2-hydroxy-2methylpropiophenone (HOMPP) photo-initiator (weight ratio of 90 : 7 : 3 for 90% ion gel and 70 : 21 : 9 for 70% ion gel) is drop-cast, and it is exposed to UV light (100 mW·cm^−2^ at 365 nm) for 5 s. Then, 90 wt% ion gel and 70 wt% ion gel are cut and pasted to the prepared nylon fibers to form source drain and gate electrodes, respectively. Silver pastes are dropped to the fibers, defining the channel length. The obtained fibers are manually attached to anchor points to create a crossbar geometry and assembled to a fiber-shaped OECT, which is connected to metallic wires using silver paint and linked to a data acquisition device. The self-powered e-textile is fabricated by the woven TENG cloth and electronic fabrics together.

### 4.3. Characterization

SEM images are taken with a Hitachi SU8020. The electrical characterizations of the fiber-shaped triboiontronic transistor and logic device are conducted with a semiconductor parameter analyzer (Agilent B1500A) in a probe station under ambient environment. Resistance measurements are carried out over longer distances along coated fibers using a two-probe measurement setup. These measurements show that uniform coating of fibers is achieved at lengths greater than 1.5 cm. The displacements of TENGs are controlled by a linear motor. The output of TENGs is characterized by a Keithley 6514 system electrometer.

## Data Availability

The data used to support the findings of this study are available from the corresponding author upon request.

## Supplementary Materials

Supplementary Materials Figure S1: the fabrication process of the fiber-shaped OECT. After coating the thin PEDOT:PSS layer on the surface of the whole nylon fiber, the electrolyte layer and electrodes are defined successively. Figure S2: (a) the schematic diagram and (b) transfer curves of the 70 wt% ion gel contacted with source and drain electrodes. (c) The schematic diagram and (d) transfer curves of the 90 wt% ion gel contacted with the gate electrode. Figure S3: the resistance of the PEDOT:PSS-coated fibers vs. length. Figure S4: the real-time (*I*-*t*) test of the fiber-shaped OECT under different gate voltages (0-2 V). Figure S5: output curves (*I*_D_-*V*_D_) of the triboiontronic fiber-shaped OECT under different contact speeds. Figure S6: output curves (*I*_D_-*V*_D_) of nine tribotronic fiber-shaped OECTs under different displacements (0-1 mm). Figure S7: the transfer curves and on/off ratios of the fiber-shaped triboiontronic electrochemical transistor under different bending angles: 0°, 30°, 60°, and 90°.
