# Supplementary material for: Fiber-Shaped Triboiontronic Electrochemical Transistor
Source: Research (Wash D C). 2021 Apr 26;2021:9840918. doi: 10.34133/2021/9840918 (PMC8098052; doi:10.34133/2021/9840918)
Supplement: Supplementary Materials — Figure S1: the fabrication process of the fiber-shaped OECT. After coating the thin PEDOT:PSS layer on the surface of the whole nylon fiber, the electrolyte layer and electrodes are defined successively. Figure S2: (a) the schematic diagram and (b) transfer curves of the 70 wt% ion gel contacted with source and drain electrodes. (c) The schematic diagram and (d) transfer curves of the 90 wt% ion gel contacted with the gate electrode. Figure S3: the resistance of the PEDOT:PSS-coated fibers vs. length. Figure S4: the real-time (I-t) test of the fiber-shaped OECT under different gate voltages (0-2 V). Figure S5: output curves (ID-VD) of the triboiontronic fiber-shaped OECT under different contact speeds. Figure S6: output curves (ID-VD) of nine tribotronic fiber-shaped OECTs under different displacements (0-1 mm). Figure S7: the transfer curves and on/off ratios of the fiber-shaped triboiontronic electrochemical transistor under different bending angles: 0°, 30°, 60°, and 90°. [file 9840918.f1.doc]

Supporting information for

Fiber-shaped Triboiontronic Electrochemical Transistor

Jinran Yu^1,2^,**^‡^**, Shanshan Qin^1^,**^‡^**, Huai Zhang^1^, Yichen Wei^1,4^, Xiaoxiao Zhu^3^, Ya Yang*^1,2,4^ and Qijun Sun*^1,2,4^

*^1^Beijing Institute of Nanoenergy and Nanosystems, Chinese Academy of Sciences, Beijing 101400, P. R. China*

*^2^School of Nanoscience and Technology, University of Chinese Academy of Sciences, Beijing 100049, P. R. China*

*^3^Beijing Institute of Fashion Technology, Beijing 100029, P. R. China*

*^4^Center on Nanoenergy Research, School of Physical Science and Technology, Guangxi University, Nanning 530004, P. R. China*

*^5^Department of Materials Science WW-4, LKO, University of Erlangen-Nuremberg, Martensstrasse 7, 91058 Erlangen, Germany*

***^‡^****Jinran Yu and Shanshan Qin contributed equally to this work.*

*Correspondence should be addressed to Qijun Sun; [sunqijun@binn.cas.cn](mailto:sunqijun@binn.cas.cn) and Ya Yang; [yayang@binn.cas.cn](mailto:yayang@binn.cas.cn)

**Supplementary Note**

*Possible humidity influences:* PEDOT: PSS has been widely used in transistors and other electronic devices, but it is also sensitive to humidity. Relative humidity (RH) has a great influence on the performance of PEDOT: PSS transistor, which has been widely studied in previous literatures. Due to the interaction between materials and adsorbed water, the device performance will be affected [1, 2]. With the increase of humidity, the performance of the device decreases. The water absorption of PSS may lead to the expansion of the film, which may also cause mechanical stress on other functional layers of organic devices[3]. The presence of water leads the resistance increase of PEDOT:PSS[4, 5], which is due to the swelling of polymer due to water absorption at high relative humidity, thus reducing the interaction between PEDOT: PSS chains. Generally, the PEDOT: PSS conductive mechanism is mainly due to the electron jumping between PEDOT chains, and the PSS chain is responsible for water absorption due to its hydrophilicity. The distance between adjacent PEDOT chains increases when the PSS chain absorbs moisture (generally the humidity increases from 10% to 70%). Therefore, the mechanism of charge jump conduction between PEDOT chains becomes more difficult, and with the increase of humidity, the its conduction will be decreased[5, 6]. Therefore, the PEDOT:PSS transistor will have a degraded performance under the moisture environment. By introducing passivation layer or hydrophobic material, the influence of humidity on PEDOT: PSS can be reduced, and the humidity stability of the device may be greatly improved [7]. On the other hand, due to the susceptibility of PEDOT:PSS to moisture, the fibers-shaped triboiontronic electrochemical device is possible to be used as a potential humidity sensor.

**Supplementary Figures**

(1) Fabrication process of the fiber-shaped OECT.


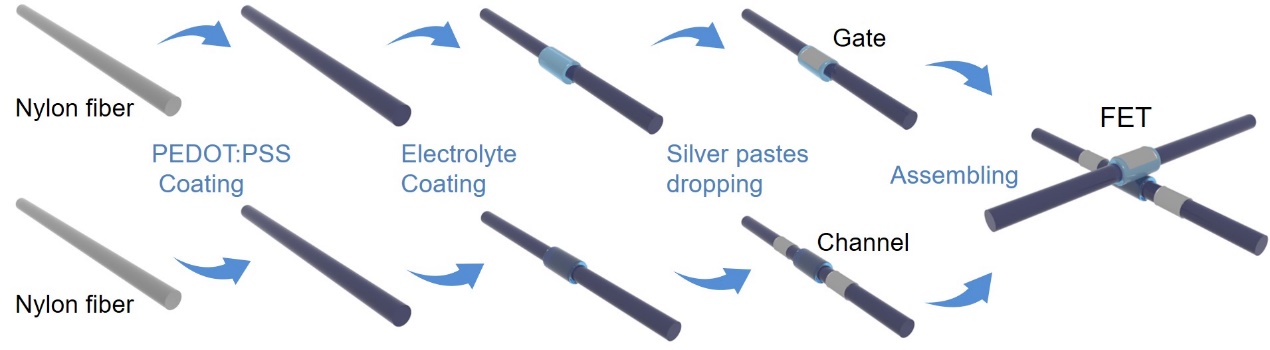


**Figure S1.** The fabrication process of the fiber-shaped OECT. After coating the thin PEDOT:PSS layer on the surface of the whole nylon fiber, the electrolyte layer and electrodes are defined successively. The obtained ﬁbers are attached to anchor points to create a crossbar geometry and assembled to a fiber-shaped OECT.

(2) Electrical performances of OECTs based on 70 wt% ion gel and 90 wt% ion gel.


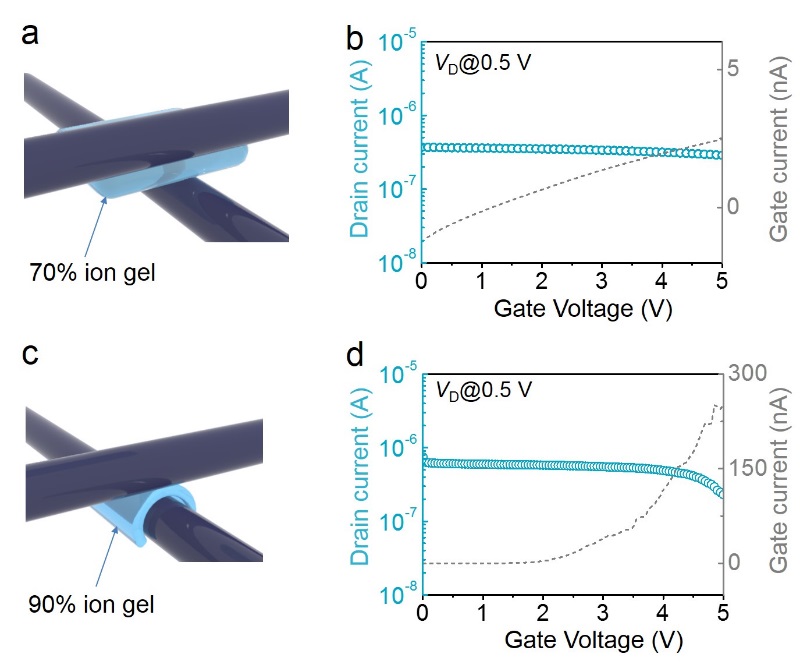


**Figure S2.** (a) The schematic diagram, and (b) transfer curves of the 70 wt% ion gel contacted with source and drain electrodes. (c) The schematic diagram, and (d) transfer curves of the 90 wt% ion gel contacted with gate electrode.

(3) Relationship between the resistance of the PEDOT:PSS-coated fibers and length.


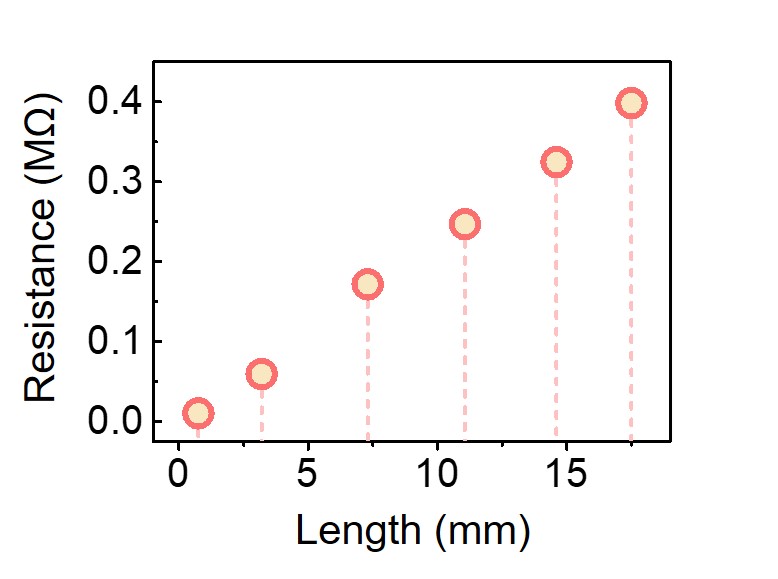


**Figure S3.** The resistance of the PEDOT:PSS-coated fibers *vs.* length.

(4) The real-time test of the fiber-shaped OECT.


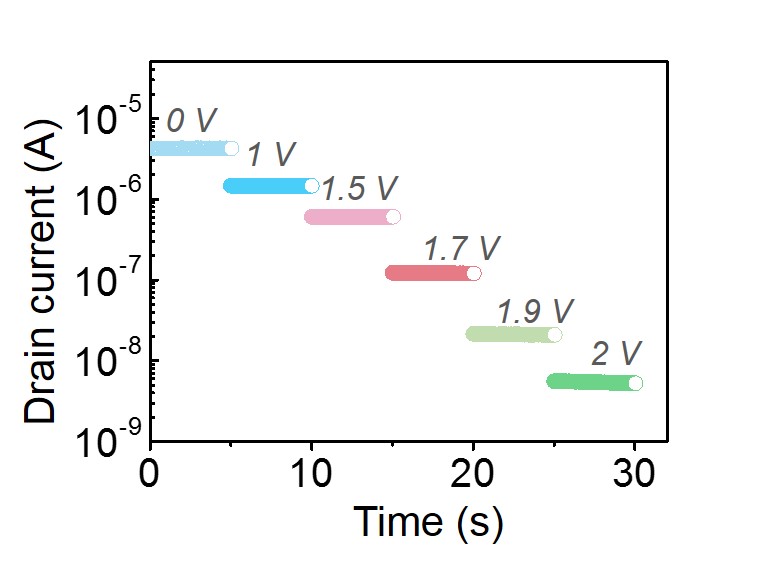


**Figure S4.** The real-time (*I-t*) test of the fiber-shaped OECT under different gate voltages (0-2 V).

(5) Output characteristics of the triboiontronic fiber-shaped OECT.


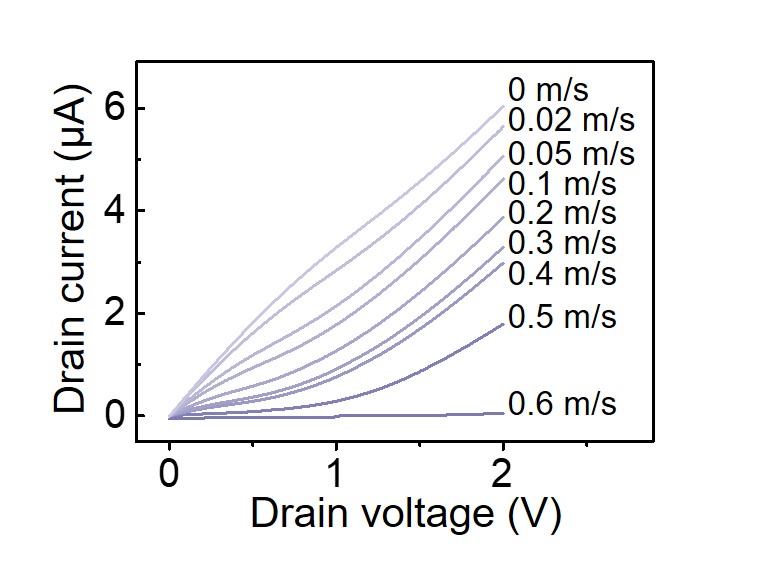


**Figure S5.** Output curves (*I*_D_*-V*_D_) of the triboiontronic fiber-shaped OECT under different contact speeds.

(6) Output characteristics of nine triboiontronic fiber-shaped OECTs.


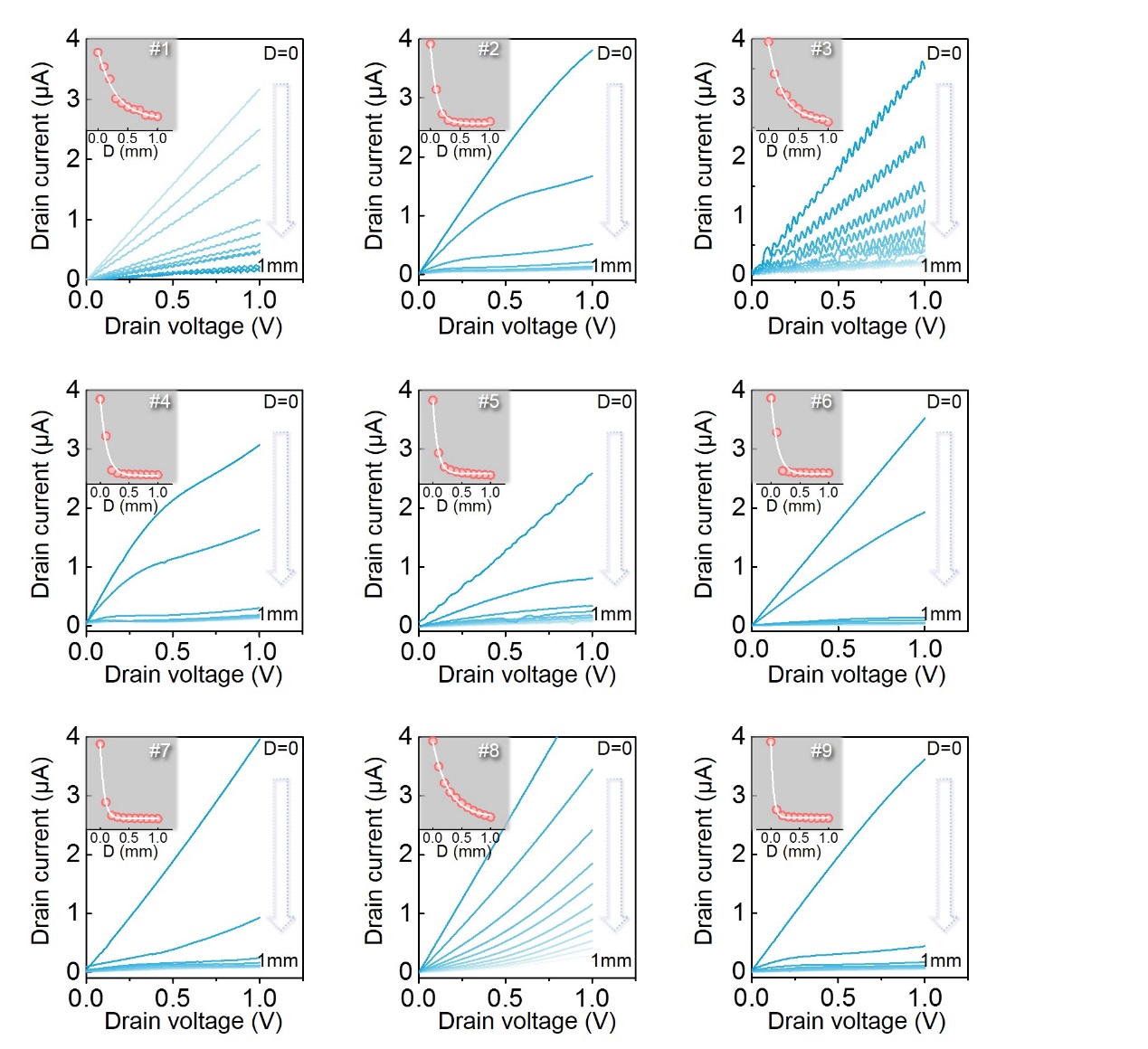


**Figure S6.** Output curves (*I*_D_*-V*_D_) of nine tribotronic fiber-shaped OECTs under different displacements (0-1 mm).

(7) Performances of the fiber-shaped triboiontronic electrochemical transistor under different bending states.


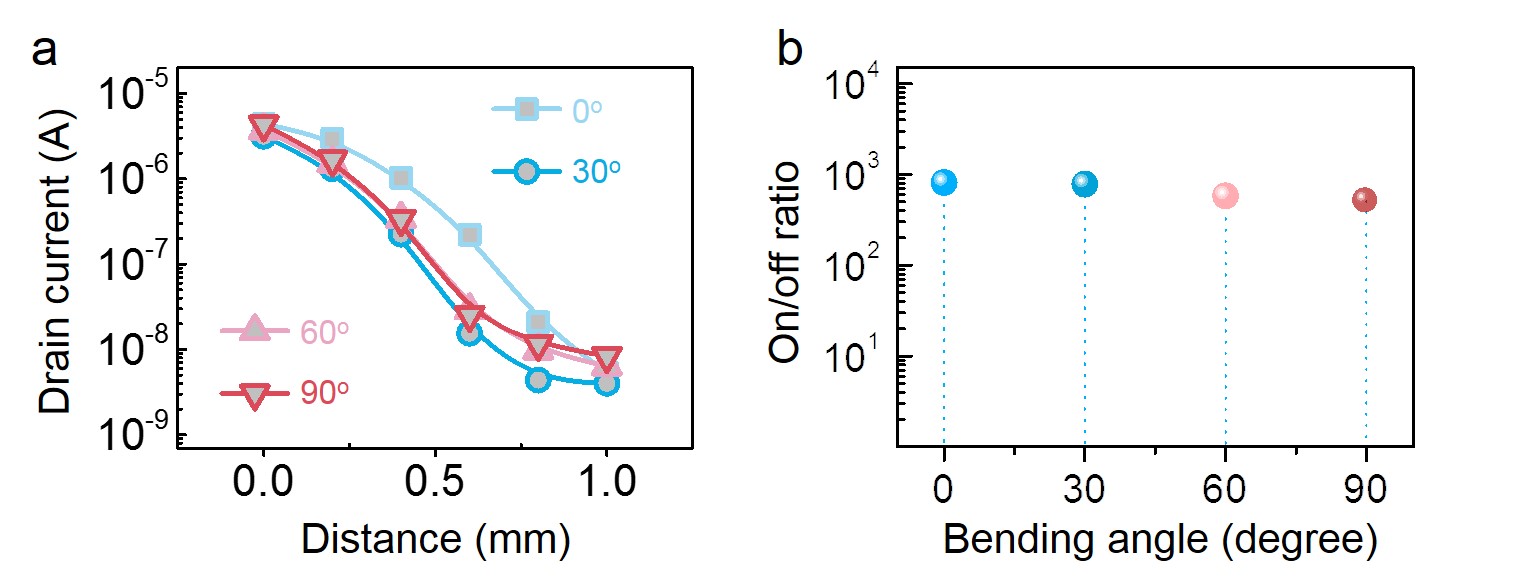


**Figure S7.** The transfer curves and on/off ratios of the fiber-shaped triboiontronic electrochemical transistor under different bending angles, 0°, 30°, 60°, 90°.

**References：**

[1] O. D. Jurchescu, J. Baas, T. T. M. Palstra, “Electronic transport properties of pentacene single crystals upon exposure to air,” *Applied Physics Letters*, vol. 87, no. 5, pp. 052102, 2005.

[2] C. Goldmann, D. J. Gundlach, B. Batlogg, “Evidence of water-related discrete trap state formation in pentacene single-crystal field-effect transistors,” *Applied Physics Letters*, vol. 88, no. 6, pp. 063501, 2006.

[3] L. Biessmann, L. P. Kreuzer, T. Widmann, et al., “Monitoring the swelling behavior of PEDOT:PSS electrodes under high humidity conditions,” *ACS Appl Mater Interfaces*, vol. 10, no. 11, pp. 9865-9872, 2018.

[4] J. Huang, P. F. Miller, J. S. Wilson, et al., “Investigation of the effects of doping and post-deposition treatments on the conductivity, morphology, and work function of poly(3,4-ethylenedioxythiophene)/poly(styrene sulfonate) films,” *Advanced Functional Materials*, vol. 15, no. 2, pp. 290-296, 2005.

[5] A. Benchirouf, S. Palaniyappan, R. Ramalingame, et al., “Electrical properties of multi-walled carbon nanotubes/PEDOT:PSS nanocomposites thin films under temperature and humidity effects,” *Sensors and Actuators B: Chemical*, vol. 224, no. pp. 344-350, 2016.

[6] M. Kus, S. Okur, “Electrical characterization of PEDOT:PSS beyond humidity saturation,” *Sensors and Actuators B-Chemical*, vol. 143, no. 1, pp. 177-181, 2009.

[7] Y. F. Wang, T. Sekine, Y. Takeda, et al., “Fully printed PEDOT:PSS-based temperature sensor with high humidity stability for wireless healthcare monitoring,” *Sci Rep*, vol. 10, no. 1, pp. 2467, 2020.
